# Integrative multi-omics analysis to gain new insights into COVID-19

**DOI:** 10.1038/s41598-024-79904-z

**Published:** 2024-11-30

**Authors:** Setegn Eshetie, Karmel W. Choi, Elina Hyppönen, Beben Benyamin, S. Hong Lee

**Affiliations:** 1 Australian Centre for Precision Health, University of South Australia, Adelaide, SA 5000 Australia; 2 UniSA Allied Health and Human Performance, University of South Australia, Adelaide, SA 5000 Australia; 3 Department of Medical Microbiology, College of Medicine and Health Sciences, University of Gondar, 196, Gondar, Ethiopia; 4 South Australian Health and Medical Research Institute (SAHMRI), University of South Australia, Adelaide, SA 5000 Australia; 5 Center for Precision Psychiatry, Department of Psychiatry, Massachusetts General Hospital, Boston, MA USA; 6 Psychiatric and Neurodevelopmental Genetics Unit, Center for Genomic Medicine, Massachusetts General Hospital, Boston, MA USA; 7 UniSA Clinical and Health Sciences, University of South Australia, Adelaide, SA 5000 Australia

**Keywords:** COVID-19, Integrative-analysis, Multi-omics interplay, Genetics, Microbiology, Diseases, Medical research

## Abstract

Multidimensional host and viral factors determine the clinical course of COVID-19. While the virology of the disease is well studied, investigating host-related factors, including genome, transcriptome, metabolome, and exposome, can provide valuable insights into the underlying pathophysiology. We conducted integrative omics analyses to explore their intricate interplay in COVID-19. We used data from the UK Biobank (UKB), and employed single-omics, pairwise-omics, and multi-omics models to illustrate the effects of different omics layers. The dataset included COVID-19 phenotypic data as well as genome, imputed-transcriptome, metabolome and exposome data. We examined the main, interaction effects and correlations between omics layers underlying COVID-19. Single-omics analyses showed that the transcriptome (derived from the coronary artery tissue) and exposome captured 3–4% of the variation in COVID-19 susceptibility, while the genome and metabolome contributed 2–2.5% of the phenotypic variation. In the omics-exposome model, where individual omics layers were simultaneously fitted with exposome data, the contributions of genome and metabolome were diminished and considered negligible, whereas the effects of the transcriptome showed minimal change. Through mediation analysis, the findings revealed that exposomic factors mediated about 60% of the genome and metabolome’s effects, while having a relatively minor impact on the transcriptome, mediating only 7% of its effects. In conclusion, our integrative-omics analyses shed light on the contribution of omics layers to the variance of COVID-19.

**Supplementary Information:**

The online version contains supplementary material available at 10.1038/s41598-024-79904-z.

## Background

Coronavirus disease 2019 (COVID-19) is a viral-induced inflammatory disease that causes acute respiratory distress syndrome (ARDS) in some patients^1,2^. Infectious diseases including COVID-19 are viewed differently from other complex diseases as they are largely determined by exposure to a microbial pathogen, with some contribution from host-related factors^3^. While prior knowledge suggests that multidimensional host, viral, and environmental factors determine the clinical course of COVID-19^4^, advances in research on the disease have been largely driven by understanding of virus biology, infection mechanisms, and virulence^5,6^, with less emphasis on host-associated and external features^7^. It is important to note that not all people exposed to the virus will develop the same disease^8,9^, and given disease heterogeneity, understanding host-associated factors is a better way to look for possible interventions.

Genetic and environmental factors greatly contribute to phenotypic variation among individuals^10,11^. While genetic studies have demonstrated associations between genetic variations and complex traits, a comprehensive mechanistic understanding of these relationships remains uncertain^12,13^. This underscores the importance of delving into how multi-omics layers interact to unravel the details of disease etiology and progression. To capture the proportion of unexplained phenotypic variation, comprehensive analysis of the genome in conjunction with other multi-omics data (transcriptome, proteome, metabolome, exposome, etc.) is required to define phenotypic variation between populations and/or individuals, to maximize etiological understanding of disease^12,14^. Several omics approaches, such as genomics, transcriptomics, proteomics, metabolomics, and exposomes, each reveal the association of corresponding omics layers with complex traits^13,15,16^.

The use of multi-omics approaches in the analysis of complex traits has been relatively limited, with only a few studies exploring its potential^12^. Zhou and Lee^17^ employed an integrative analysis of genomic and exposomic data that enabled to capture a greater proportion of phenotypic variance for anthropometric traits such as body mass index (BMI) and height, resulting in improved accuracy in phenotypic prediction, compared to the genomic data alone. The authors also highlighted that both additive and non-additive effects of multi-omics contribute to shaping the phenotypic variance. Similarly, another integrative analysis of genome and exposome^18^ focused on mental health was applied, showing that in addition to the main effects, genome-exposome interactions played a significant role in mental health traits, including internalizing and externalizing symptoms. Moreover, researchers have now expanded the integrative multi-omics approach by incorporating additional layers of omics data such as transcriptomics, proteomics, metabolomics, lipidomics and methylomics. These comprehensive analyses have been used to dissect the influence of both genetic and environmental factors on various diseases like Type 2 diabetes, osteoarthritis, cardiovascular diseases, Alzheimer’s disease and systemic lupus erythematosus^19–22^.

Attempts have been made to explore the host genetic basis of COVID-19 through genome-wide association studies^23,24^, but the genome still captures the few portions of the disease phenotypic variance and little attention has been paid to other biological facets. Virus-host relationships are complex and difficult to fully characterize, so integrated multiple genome-wide omics approaches may be useful. Few studies have attempted to examine the potential role of molecular signatures (e.g., genome and transcriptome) and clinical covariates in association with the COVID-19 phenotype, but the results are either based on individual omics analyses or on the integration of a few omics layers^25,26^. The integrative analysis of multi-omics data may also enhance the understanding of the molecular dynamics underlying the pathophysiology of COVID-19, and may lead to novel strategies for early detection, prevention, and treatment of the disease.

In this study, we conducted a comprehensive multistage integrative multi-omics analysis covering genome, imputed gene-expression levels (transcriptome), metabolome and exposome (Supplementary note 1), to explore the interplay among these factors and their effects on COVID-19. We first estimated both additive and non-additive variance components for each omics layer, i.e. quantifying individual omics contribution to COVID-19 phenotypic variation. We also explored the correlations and interactions between individual omics effects on COVID-19. For the purpose of quantifying interaction and correlation effects, we applied a novel linear mixed model known as CORE-REML^27^, which can handle multiple variance-covariance structures and explicitly estimates the covariance between random effects. By comprehensively examining these multiple omics layers, we aimed to gain insights into the multi-omics data underlying COVID-19, uncover novel biomarkers, and understand the interrelationships between genetic, transcriptomic, metabolic, and environmental factors.

## Methods

### Ethics declarations

We used data from the UK Biobank (UKB) for our analyses. The UKB has approval from the Northwest Multi-centre Research Ethics Committee (MREC), National Information Governance Board for Health & Social Care (NIGB), and Community Health Index Advisory Group (CHIAG) (http://www.ukbiobank.ac.uk/ethics/*).* UKB has obtained informed consent from all participants. These ethical regulations cover the work in this study and our accession to the UKB data was under the reference number 14,575. All methods were performed in accordance with the relevant guidelines and regulations.

### Phenotypic data and case definition

The present study used both phenotypic and genotypic data from UKB participants who underwent COVID-19 testing. Based on their test results, we classified participants into three categories: those who tested negative, those with infection presenting with moderate symptoms, and those with infection presenting with severe symptoms^28,29^. Severe cases were defined as individuals who received a positive clinical diagnosis (acute respiratory distress syndrome, sepsis, septic shock, and etc.) of COVID-19 and required hospital admission, intensive care unit admission with respiratory support (either non-invasive or invasive ventilation) or died from COVID-19 as the primary cause of death. In contrast, moderate cases were defined as individuals who received a clinical diagnosis of COVID-19 and were either inpatients or outpatients not requiring respiratory support, clinically diagnosed individuals in the general population, or self-reported quarantined individuals. Taking into account the clinical course of the infection, we defined the COVID-19 phenotype according to the ordinal disease classification proposed by the World Health Organization (WHO)^28^. Therefore, controls were COVID-19-negative individuals (coded 0), and cases (who tested positive for COVID-19) were further divided into moderate cases (coded 1) and severe cases (coded 2) based on clinical severity scores.

### UK Biobank and population

The UKB is a population-based cohort of over 500,000 people (aged 40 to 69 years at recruitment) recruited from England, Scotland and Wales between 2006 and 2010. UKB provides comprehensive baseline measurements/questionnaires, biomarkers, demographic characteristics (e.g., age, gender, socioeconomic status, etc.) and longitudinal clinical phenotypes (cancer, death, hospitalization, etc.). UKB has also recently made available COVID-19 research data (including test results, death registrations, hospital admissions, primary care, and other data). As of March 3, 2022, a total of 434,119 COVID-19 tests have been conducted, of which nearly 9.5% (40,949) were positive results. A total of 144,278 people were tested for COVID-19, of whom 28,003 tested positive during the period. Specifically, this study analysed the largest ancestry group, the British white population (408,183 participants), to minimize genetic heterogeneity (Supplementary note 2).

### Study design

This study aimed to investigate the extent to which genetic and non-genetic factors contribute to COVID-19 phenotypic variation. Specifically, we analysed the genome, transcriptome, metabolome, and exposome to understand their respective impacts. The details on the phenotypic data and each omics signature are provided below.

**Fig. 1:**
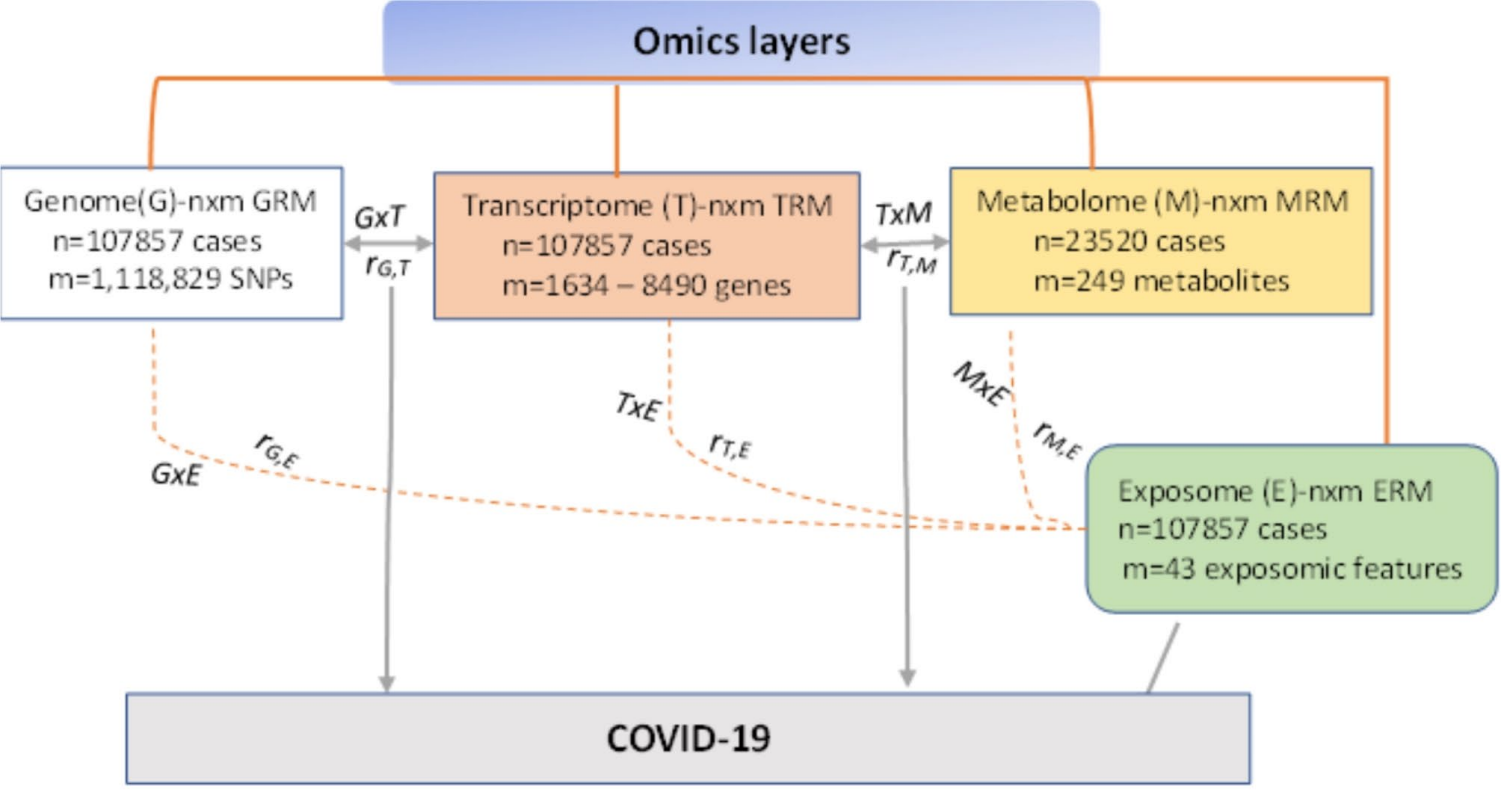
A diagram of study design for partitioning of COVID-19 phenotypic variance using multi-omics data. The phenotypic variance partitioning analyses were based on multi-omics layers, including genome (G), transcriptome (T), metabolome (M) and exposome (E). Phenotypic variance also captured by omics-exposome interactions, such as genome-exposome (GxE), transcriptome-exposome (TxE), and metabolome-exposome (MxE). Interactions between omics layers, genome-transcriptome (GxT), and transcriptome-metabolome (TxM) were also explored to examine variance components. Furthermore, it is worth noting that due to complex interplays between omics layers, part of the phenotypic variation can be explained by covariance structures, thus revealing omics correlations. Correlation structures were sought between genome & exposome (r_G,E_), genome & transcriptome (r_G,T_), transcriptome & exposome (r_T,E_), transcriptome & metabolome (r_T,M_), and metabolome & exposome (r_M,E_). Apart from metabolomics data (only 23520 samples were accessed), other omics data were obtained from 107857 UKB participants. Genome and metabolome interplay was not carried out because the effect of the genome was negligible, based on the effect of metabolome-matched cases on phenotypic variation.

As shown in Fig. 1, our analysis began by estimating the single nucleotide polymorphism-based heritability (SNP-h^2^) of COVID-19 based on host genome information. Next, we explored the contributions of imputed transcriptome (tissue gene expression levels), biomarkers (including lipids and amino acids, and etc.), and exposomic characteristics (socio-demographic data, physical measures, behavioural factors, population structure to account for genetic similarity, diet intake, and medical conditions) using a linear mixed model. Additionally, we investigated the interaction effects of the exposome with the genome, transcriptome, metabolome, and exposome on COVID-19 (Fig. 1). This design allowed us to identify potential gene-environment interactions that could impact COVID-19 susceptibility and severity. Overall, this multi-omics approach allowed us to explore the complex interplay between genetic and non-genetic factors in COVID-19 phenotypic variation and identify potential targets for further research and intervention.

### Genotypic data and quality control

**Fig. 2:**
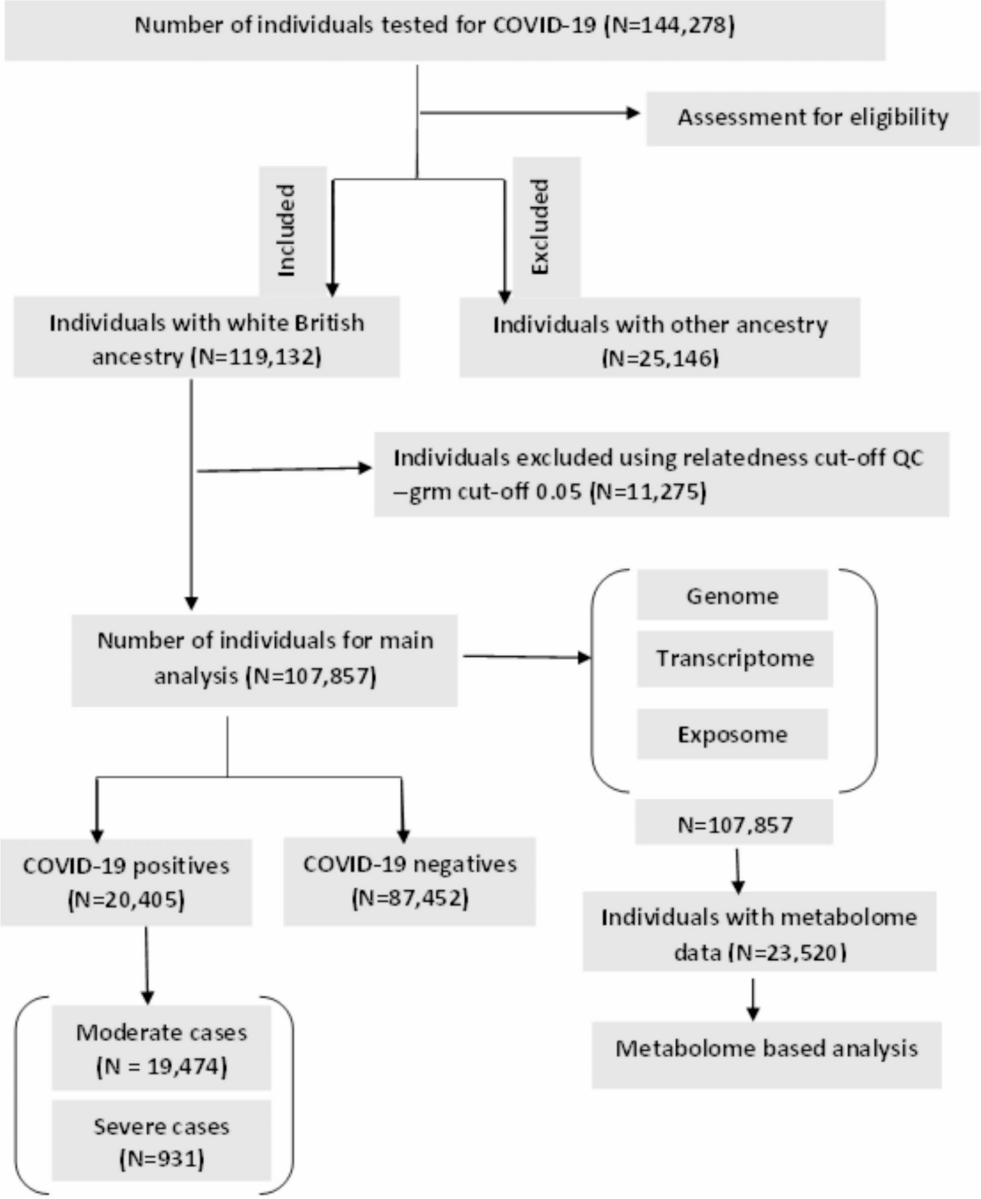
Flow chart showing the summary of UKB COVID-19 positive and negative participants and the selection procedure of study subjects for inclusion in the study.

This study analysed individuals who were of White British ancestry (119,132 individuals) to minimise the effects of population stratification. Quality controls (QC) procedures have been carried out at the SNP and individual levels, and a total of 7,701,772 SNPs and 107,857 individuals remained for further analyses. For SNP-level QC, variants with info score < 60%, multi-character allele codes, minor allele frequency (MAF) < 1%, the Hardy-Weinberg equilibrium (HWE) *P* < 1e-7, SNP call rate < 95% and duplicate ID variants were excluded in down-stream analyses. For individual-level QC, individuals with a missing rate of genotype > 5%, gender mismatch, poor genotype quality or a sex chromosome aneuploidy, and non-white British subjects were excluded from the main analyses. Furthermore, we assessed the genetic relatedness between a pair of individuals after constructing a genetic relationship matrix (GRM) and applied relatedness cut off QC (> 5%), using genome-wide complex trait analysis (GCTA)^30^. Thus, out of a total of 144,278 COVID-19 test subjects, 119,132 were retained according to the quality control steps described above, and 107,857 (57362 females, 50495 males) were finally considered for downstream analysis after removal of pairwise relationship > 5% (Fig. 2). To estimate SNP-h^2^, we extracted 1,118,829 SNPs from the HapMap3 database, which is known for its high-quality SNPs^17,31^, aiming to improve computational performance and reliability.

### Transcriptomic data and imputation of gene expression levels

To better understand the genetic factors underlying COVID-19, we utilized imputation to estimate genetically predicted gene expression levels using individual genotype data^27,32–35^. This process was carried out using MetaXcan, which is an updated version of PrediXcan. Specifically, we used tissue-specific elastic net models that incorporated around 200,000 cis-expression quantitative trait loci (cis-eQTLs) based on GTEx v8, the reference transcriptome dataset.

We generated transcriptome data for 18 tissues (e.g., coronary artery, whole blood, spleen and etc.) that were selected based on their potential roles in the pathogenesis of the disease^36–38^. We estimated gene expression levels for almost all genes across 18 tissues and used approximately 90% of eQTLs (SNPs) in COVID-19 genotype data to predict transcriptome information (Supplementary note 3: Table S2-S3).

### Metabolomic data

Our study utilized metabolomic biomarkers consisting of 249 metabolites from 118,461 individuals from UKB^39^. These metabolites were measured in preserved blood samples collected from the UKB cohort between 2006 and 2010. This dataset provided information on the plasma concentration levels of circulating lipids, lipoprotein subclasses, fatty acid composition, and various other low-molecular metabolites. However, we only had access to metabolome data for 23,520 COVID-19 cases.

To measure these biomarkers, the UK Biobank used a high-throughput NMR-based metabolic biomarker profiling platform to analyse randomly selected EDTA plasma samples (aliquot 3). This allowed them to measure the metabolomic biomarkers efficiently and accurately in our study participants.

### Exposomic data

Our analysis incorporates a comprehensive set of exposomic characteristics, including socio-demographic and population structure, dietary intake, and medical conditions.

These features were selected based on prior research that underscores their significant role in influencing susceptibility to COVID-19, as evidenced by the existing literature (Table 1). We collected and analysed exposomic data from 107,857 participants in the UK Biobank, along with their associated COVID-19 data. We evaluated the entirety of all exposomic features by constructing a single cohesive framework or variance-covariance matrix for assessing their global effect on COVID-19 susceptibility. Additionally, we assessed the individual effect of each exposomic variable on COVID-19 (Table S4).

**Table 1:** Description of selected features used to capture the exposome.

Exposomic features	Description
Socio-demographic^40,41^, physical measures^42,43^, behavioural factors^40,44^, and population structure^7,45^	Age at recruitment, year of birth, sex, Townsend deprivation index, education status, body mass index, hip circumference, sitting height, standing height, waist circumference, weight, alcohol use, smoking and ancestry principal components (PCs)
Dietary intake^46–48^	Bread intake, cereal intake, coffee intake, cooked vegetable intake, dried fruit intake, fresh fruit intake, salad/ raw vegetable intake, tea intake, water intake, beef intake, cheese intake, lamb/mutton intake, oily fish intake, pork intake and poultry intake
Medical conditions^49–51^	Cancer diagnosed by doctor, diabetes diagnosed by doctor, blood clot, deep vein thrombosis (DVT), bronchitis, emphysema, asthma, rhinitis, eczema, allergy diagnosed by doctor, diastolic blood pressure automated reading, pulse rate automated reading, and systolic blood pressure automated reading

### Modelling and data analysis

We first estimated gene expression levels in 18 tissues using MetaXcan at the individual SNP level. The estimated tissue-specific transcriptome association signal with COVID-19 was then assessed using multiple linear regression. Five tissue models (e.g., coronary artery, whole blood, spleen, musculoskeletal, and adipose viscera) were selected based on the significance level and used for downstream comprehensive analysis. Then, generalized linear models were used to adjust the COVID-19 phenotype for fixed effects (genotype measurement batch and UKB assessment centre) before applying the linear mixed model framework. We also constructed GRM with the 1,118,829 SNPs based on the HapMap3 SNPs, transcriptomic relation matrix (TRM) with tissue-specific gene expression levels (1634–8490 genes), metabolomic relationship matrix (MRM) with 249 metabolites, and exposomic relationship matrix (ERM) with 43 exposomic features. We also generated matrices based on interaction or correlation between each pair of omics layers. We estimated the variance components due to additive and interaction effects of omics using advanced linear mixed model based on genome-based restricted maximum likelihood (GREML). Additionally, we applied CORE-GREML to estimate correlations between random omics effects. COVID-19 phenotypic variances explained by genome, transcriptome, metabolome, exposome, interaction, and covariance between pairs of omics layers are described below (Table 2). Given the sample size of the omics datasets, the variance component analysis offered adequate statistical power to capture the proportion of phenotypic variance (https://shiny.cnsgenomics.com/gctaPower/)^52^ (Figure S2). Data analyses were conducted using MTG2 (linear mixed models including CORE-GREML)^53^, PLINK version 2^54^, MetaXcan (transcriptome imputation)^55^, and R-programming (data manipulation and visualization).

Additionally, mediation analysis was conducted to explore the pathways through which independent variables including genomic, transcriptomic, and metabolomic data affect the dependent variable COVID-19, via a mediator variable (MV), the exposome. This analysis utilized omics-specific risk scores^56–58^, including genomic, transcriptomic, metabolomic, and exposomic risk scores. These scores were derived by splitting the data into an 80% discovery set and a 20% test set to avoid overfitting, and were implemented in MTG2^53^. To ensure result robustness, five-fold cross-validation^59–61^ was applied, and average estimates were used to assess the exposome’s mediating effect on the genome, transcriptome, and metabolome in relation to COVID-19 susceptibility. Individual based risk scores from genome, transcriptome, metabolome, and exposome datasets were loaded, and linear regression models were fitted to estimate the relationships between these variables. Mediation analysis was conducted with the R mediation package^61^ to estimate the average causal mediation effect (ACME), direct effect (DE), total effect and the proportion of mediation. Bootstrapping was used to obtain robust confidence intervals for the indirect effects.

**Table 2:** Description of multi-omics models for estimating variance components.

Analysis scope	Model	Description
Model 1. Single-omics analysis: estimate variance components using omics layers separately	$\mathbf{Y}=\mathbf{E}+\varvec{\upepsilon}$	$\mathbf{Y}$ is the vector of COVID-19 outcome, $\mathbf{G}$ is the vector of genome effect, and $\varvec{\upepsilon}$ is the vector of residuals, $\mathbf{T}$ is the vector of transcriptome effect, $\mathbf{M}$ is the vector metabolome effect, $\mathbf{E}$ is the vector of exposome effect. **G**$\mathbf{x}\mathbf{E}$ is the vector of gene by environment interaction, **T**$\mathbf{x}\mathbf{E}$ is the vector of transcriptome by environment interaction, **M**$\mathbf{x}\mathbf{E}$ is the vector of metabolome by environment interaction. **r**$\mathbf{G},\mathbf{E}$ is the vector of covariance between genome and exposome, **r**$\mathbf{T},\mathbf{E}$ is the vector of covariance between genome and exposome, and **r**$\mathbf{M},\mathbf{E}$ is the vector of covariance between metabolome and exposome. **G**$\mathbf{x}\mathbf{T}$ is the vector of genome by transcriptome interaction, **r**$\mathbf{G},\mathbf{T}$ is the vector of covariance between genome and transcriptome. **T**$\mathbf{x}\mathbf{M}$ is the vector of transcriptome by metabolome interaction, **r**$\mathbf{T},\mathbf{M}$ is the vector of covariance between transcriptome and metabolome.
	$$\mathbf{Y}=\mathbf{T}+\varvec{\upepsilon}$$	
	$$\mathbf{Y}=\mathbf{M}+\varvec{\upepsilon}$$	
	$$\mathbf{Y}=\mathbf{G}+\varvec{\upepsilon}$$	
Model 2: Pairwise omics analysis: joint analysis of genome, transcriptome, and metabolome	$\mathbf{Y}=\mathbf{G}+\mathbf{T}+\varvec{\upepsilon}$	
	$$\mathbf{Y}=\mathbf{G}+\mathbf{M}+\varvec{\upepsilon}$$	
	$$\mathbf{Y}=\mathbf{G}+\mathbf{E}+\varvec{\upepsilon}$$	
	$$\mathbf{Y}=\mathbf{T}+\mathbf{M}+\varvec{\upepsilon}$$	
	$$\mathbf{Y}=\mathbf{T}+\mathbf{E}+\varvec{\upepsilon}$$	
	$$\mathbf{Y}=\mathbf{M}+\mathbf{E}+\varvec{\upepsilon}$$	
Model 3. Joint model fitting each omics data type with exposome	$\mathbf{Y}=\mathbf{G}+\mathbf{E}+\mathbf{G}\mathbf{x}\mathbf{E}+\varvec{\upepsilon}$	
	$$\mathbf{Y}=\mathbf{T}+\mathbf{E}+\mathbf{T}\mathbf{x}\mathbf{E}+\varvec{\upepsilon}$$	
	$$\mathbf{Y}=\mathbf{M}+\mathbf{E}+\mathbf{M}\mathbf{x}\mathbf{E}+\varvec{\upepsilon}$$	
	$$\mathbf{Y}=\mathbf{G}+\mathbf{E}+\mathbf{r}\mathbf{G},\mathbf{E}+\mathbf{G}\mathbf{x}\mathbf{E}+\varvec{\upepsilon}$$	
	$$\mathbf{Y}=\mathbf{T}+\mathbf{E}+\mathbf{r}\mathbf{T},\mathbf{E}+\mathbf{T}\mathbf{x}\mathbf{E}+\varvec{\upepsilon}$$	
	$$\mathbf{Y}=\mathbf{M}+\mathbf{E}+\mathbf{r}\mathbf{M},\mathbf{E}+\mathbf{M}\mathbf{x}\mathbf{E}+\varvec{\upepsilon}$$	
Model 4. Joint models fitting genome and transcriptome and their interplay	$\mathbf{Y}=\mathbf{G}+\mathbf{T}+\varvec{\upepsilon}$	
	$$\mathbf{Y}=\mathbf{G}+\mathbf{T}+\mathbf{E}+\varvec{\upepsilon}$$	
	$$\mathbf{Y}=\mathbf{G}+\mathbf{T}+\mathbf{G}\mathbf{x}\mathbf{T}+\varvec{\upepsilon}$$	
	$$\mathbf{Y}=\mathbf{G}+\mathbf{T}+\mathbf{r}\mathbf{G},\mathbf{T}+\varvec{\upepsilon}$$	
	$$\mathbf{Y}=\mathbf{G}+\mathbf{T}+\mathbf{G}\mathbf{x}\mathbf{T}+\mathbf{E}+\varvec{\upepsilon}$$	
	$$\mathbf{Y}=\mathbf{G}+\mathbf{T}+\mathbf{G}\mathbf{x}\mathbf{T}+\mathbf{r}\mathbf{G},\mathbf{T}+\mathbf{E}+\varvec{\upepsilon}$$	
Model 5. Joint models fitting transcriptome and metabolome and their interplay	$\mathbf{Y}=\mathbf{T}+\mathbf{M}+\varvec{\upepsilon}$	
	$$\mathbf{Y}=\mathbf{T}+\mathbf{M}+\mathbf{E}+\varvec{\upepsilon}$$	
	$$\mathbf{Y}=\mathbf{T}+\mathbf{M}+\mathbf{T}\mathbf{x}\mathbf{M}+\varvec{\upepsilon}$$	
	$$\mathbf{Y}=\mathbf{T}+\mathbf{M}+\mathbf{r}\mathbf{T},\mathbf{M}+\varvec{\upepsilon}$$	
	$$\mathbf{Y}=\mathbf{T}+\mathbf{M}+\mathbf{T}\mathbf{x}\mathbf{M}+\varvec{\upepsilon}$$	
	$$\mathbf{Y}=\mathbf{T}+\mathbf{M}+\mathbf{T}\mathbf{x}\mathbf{M}+\mathbf{r}\mathbf{T},\mathbf{M}+\mathbf{E}+\varvec{\upepsilon}$$	

Note: An integrated omics model of the genome, transcriptome, and exposome of COVID-19 was analysed using data from 107,857 participants, but the sample size was reduced to 23,520 to account for the proportion of cases with metabolomics data.

## Results

### The effects of single omics on COVID-19 phenotypic variance

**Fig. 3:**
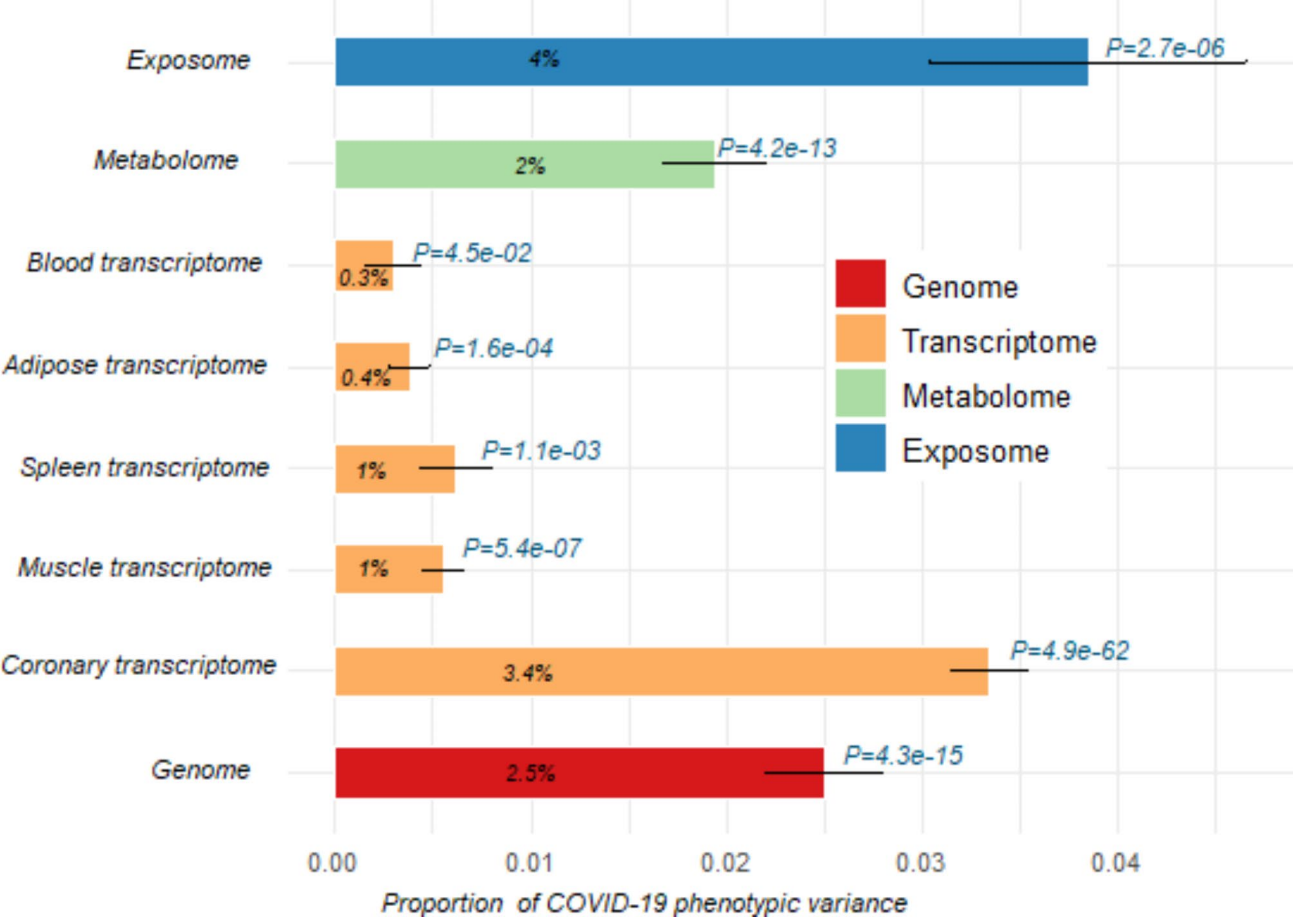
Contribution of individual omics data to COVID-19 phenotypic variance based on single-effect linear mixed models. The omics data include genome (G), transcriptome (T), metabolome, and exposome (E), and tissue-specific transcriptome data from coronary (C_T_), muscle (M_T_), spleen (S_T_), adipose (A_T_), and blood (B_T_) tissues. Exposome contributes the highest proportion to the phenotypic variance (4%), followed by imputed gene expression levels in coronary artery tissue (3.4%) and genome (2.5%). The x-axis represents the proportion of COVID-19 phenotypic variance explained by each omics data, while the y-axis represents the different tissues and omics data.

We first investigated the contributions of individual omics layers to the phenotypic variation of COVID-19 status, using a single-effect linear mixed model that fits each omics at a time. We found that genomic factors (SNP-h^2^) explained 2.5% (Standard error [Se] = 0.3%, P-value = 4.3e-15) of the phenotype variation (Fig. 3).

For transcriptomic effects, we identified significant effects in only 5 tissues: adipose viscera, coronary arteries, musculoskeletal, spleen, and whole blood. Interestingly, the highest proportion of phenotypic variation explained by imputed gene expression was found in coronary tissue at 3.4% (Se = 0.2%, P-value = 4.97e-62), whereas the effect of transcriptome in other tissues remained below 1% (Fig. 3).

In addition to gene expression, we also separately examined the contribution of metabolomic and exposomic data to COVID-19 phenotypic variation, estimated at 2.0% (Se = 0.3%, P-value = 4.2e-13) and 4.0% (Se = 0.8%, P-value = 2.7e-06), respectively (Fig. 3). Overall, exposome had the highest proportion of contribution to the phenotypic variation, followed by the coronary transcriptome, genome, and metabolome. Conversely, gene expression levels in musculoskeletal, visceral adipose, and whole blood had the least impact on COVID-19 phenotypic variation.

### Pairwise cross-omics analysis on COVID-19

**Fig. 4:**
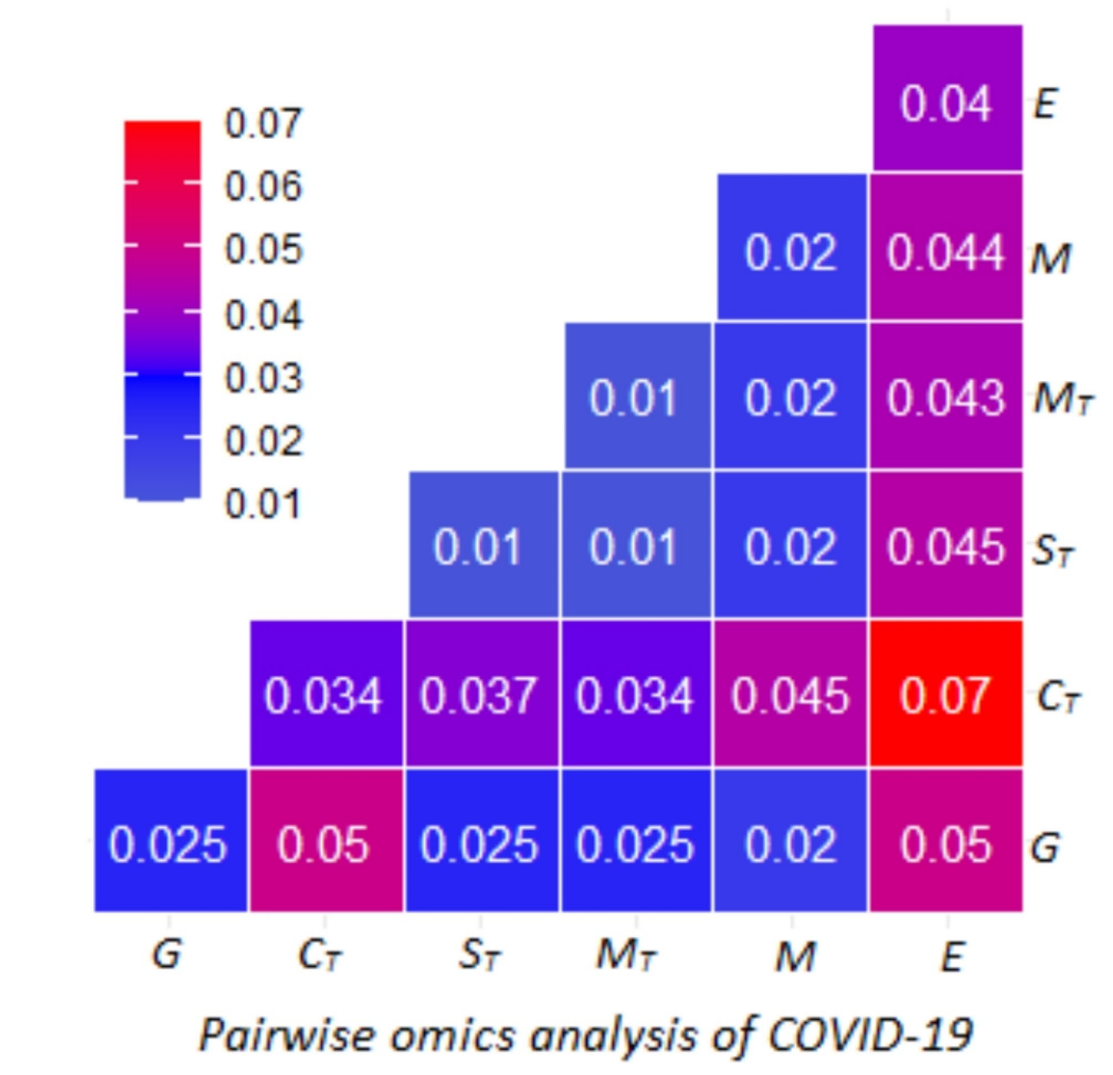
Contribution of pairwise omics to COVID-19 phenotypic variance. Pairwise omics analyses of COVID-19 were based on genome (G), coronary transcriptome (C_T_), spleen transcriptome (S_T_), muscle transcriptome (M_T_), metabolome (M) and exposome (E). In the analyses, we used a multi-effects linear mixed model that simultaneously fits pairwise omics layers to examine which parts of omics had a significant effect on COVID-19. In general, fitting expsomic and coronary transcriptomic data captures a larger proportion of COVID-19 phenotypic variance. The diagonal elements are the estimates from the single omics analysis, while the off-diagonals indicate the estimates from the pairwise omics analyses.

To gain a comprehensive understanding of phenotypic variance in COVID-19, we conducted a pairwise omics analysis to determine the contribution of each pair of omics to the phenotypic variance. Our analyses, illustrated in Fig. 4, demonstrates that combining transcriptome data from the coronary artery tissue and exposome in the model can capture nearly 7% of the COVID-19 phenotypic variation. Joint models that incorporate the genome and exposome, as well as the genome and transcriptome of the spleen tissue, explain 5% of the phenotypic variance each. We also found that the transcriptome data of the muscle skeletal tissue and metabolome significantly contribute to COVID-19, explaining 4.5% of the phenotypic variance. However, incorporating transcriptomes of other tissues with the genome did not increase the proportion of phenotypic variance explained, as compared to the genome alone. These findings suggest that analysing the exposome in conjunction with other omics layers can provide valuable insights into understand host-related etiologic factors of COVID-19, explaining up to 7% of the phenotypic variance, compared to other pairs of omics. Therefore, to estimate the extent to which the effects of omics layers are mediated by the exposome, we specifically analysed the omics layers (genome, transcriptome, and metabolome) influenced by environmental factors. By fitting multiple omics data types and exposomic effects in the same model, we can better comprehend how each omics layer contributes to COVID-19 phenotypic variance.

### Omics effects on COVID-19 mediated by exposome

**Fig. 5:**
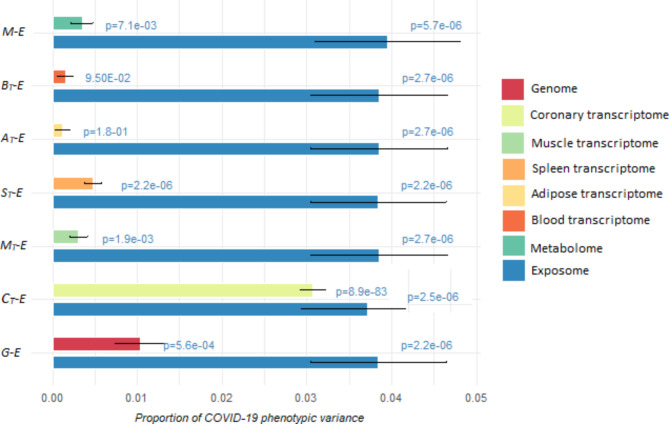
Contribution of omics data and exposome to COVID-19 phenotypic variance. The joint model fitting each omics data type with exposome estimated the relative contributions of each omics type, such as genome (G), transcriptome (T), and metabolome (M), while simultaneously accounting for the impact of environmental factors (E) on COVID-19 phenotypes, using a multi-effects linear mixed model. The x-axis represents the proportion of COVID-19 phenotypic variance explained by each omics data and by exposome data, and the y-axis represents different models including the genome-exposome (G-E), multiple tissue transcriptome-exposome (C_T_-E; M_T_-E; S_T_-E; A_T_-E; B_T_-E), and metabolome-exposome (M-E) models. The transcriptome-exposome models are based on tissue-specific gene expression profiles and exposome data, including coronary transcriptome-exposome (C_T_-E), muscle transcriptome – exposome (M_T_-E), spleen transcriptome-exposome (S_T_-E), adipose transcriptome -exposome (A_T_-E), and blood transcriptome (B_T_-E).

In Fig. 5, we present the results of our study regarding the phenotypic variance partitioning for COVID-19. Specifically, we investigated the interplay between the exposome and each of the genome, transcriptome, and metabolome in a combined model. Our analysis revealed that the additive effect of the exposome was relatively constant (~ 4%, *p* = 2.7e-06) across all omics combinations. Interestingly, we found that the genomic effect from the joint model fitting genome and exposome (G-E) was modest (~ 1%, *p* = 5.6e-04), while the metabolomic effect was also reduced but still significant (~ 0.4%, *p* = 3.5e-03). To further explore the role of the exposome in mediating gene expression, we also investigated the impact of exposome jointly along with transcriptome effects in each of five tissues (coronary arteries, spleen, musculoskeletal, whole blood, and adipose viscera). Our results showed that the additive effects of transcriptome in whole blood (~ 0.2%, *p* = 9.5e-02) and adipose visceral (~ 0.1%, *p* = 1.8e-01) became non-significant from joint models, although they exhibited sizeable significant signals in the single omics models (Fig. 3). However, we observed no marked changes in the effects of transcriptome in the other tissues (ranging from 3.4 to 3.1% for the coronary arteries, 0.6–0.5% for the spleen, and 0.6–0.5% for the musculoskeletal) upon considering the exposome layer in the model.

**Fig. 6:**
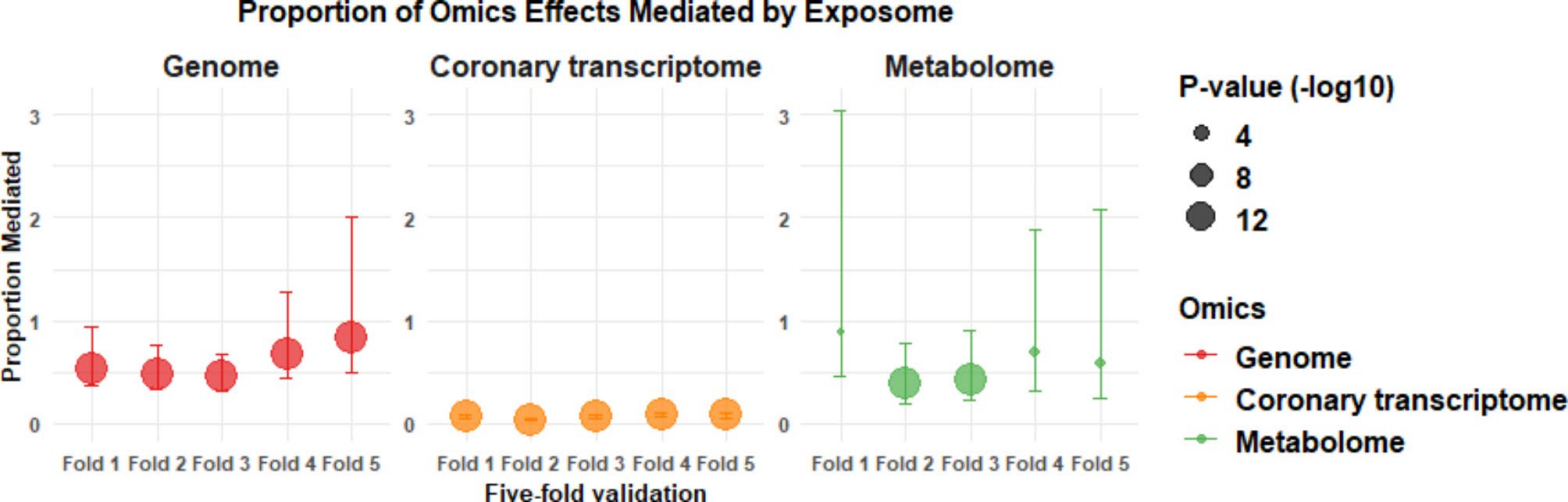
Mediation analysis of omics effects on COVID-19 Susceptibility by exposomic Factors. The five-fold cross-validation analysis (folds 1 through 5) was employed to determine the range of mediation effects. The mediation proportions represent the degree to which exposomic factors influence the relationship between each omics layer and susceptibility to COVID-19. The figure illustrates the proportion of the effects of genomic, coronary transcriptome, and metabolomic factors on COVID-19 susceptibility that are mediated by exposomic factors (left to right, respectively). Each fold presents the proportion of mediation along with 95% confidence intervals (CIs).

Supporting the results of omics-exposome models, we investigated how exposomic factors mediate the effects of the genome, transcriptome, and metabolome on COVID-19 susceptibility, with results validated through five-fold cross-validation (Fig. 6). Our findings reveal that exposomic factors significantly mediate the relationship between these omics layers and COVID-19 risk. Specifically, exposomic factors accounted for 46–83% of the genomic effects on COVID-19, with an average mediation effect of about 60%. This indicates a substantial impact of environmental and lifestyle factors on genetic susceptibility to the disease. Similarly, exposomic factors mediated 40–89% of the effects of the metabolome on COVID-19, with an average of approximately 60%, underscoring their crucial role in influencing metabolomic responses. In contrast, the mediating effect of exposomic factors on the transcriptome, particularly in coronary tissues, was relatively minor, accounting for only 4–8% (average 7%) of the transcriptomic impact on COVID-19. This suggests that while exposomic factors significantly influence genomic and metabolomic pathways, their effect on transcriptomic pathways, especially in coronary tissues, is limited. Mediation analysis for other tissue transcriptomes (such as adipose, blood, muscle, and spleen) was not performed due to their minimal impact on COVID-19.

Furthermore, we aimed to capture non-additive effects, including interactions and correlations among the omics datasets in relation to COVID-19; however, most estimations yielded no significant signals (Tables S5 and S6). Significant estimates were observed only between the genome and transcriptome (Figure S3-S4, & Table S9). Our analysis showed that the joint model, which incorporated the exposome and one molecular layer (genome, transcriptome, and metabolome) at a time, outperformed the single-omics models in capturing phenotypic variation, as demonstrated by higher model-fits (Table S7). Furthermore, paired-omics models provided accurate estimates compared to individual-only models (Table S8).

### Analysis of transcriptome, metabolome and exposome for COVID-19

We also extended the multi-omics analysis by integrating transcriptomic, metabolomic, and exposome data from matched samples, aiming to measure the variance component of COVID-19. It is noted that due to the limited availability of metabolomics data (nearly 20% of samples), genomic information had to be excluded from the analysis because genomic effects were deemed insignificant given this reduced samples. Instead, by integrating the three layers into the multi-omics model, we sought to conduct variance partitioning analysis on COVID-19 (Table S10). The results demonstrated a strong exposome effect (~ 4% of phenotypic variance), with a slight reduction in the coronary transcriptome effect from 3.4 to 2.4%. The metabolomic data contributed a marginal effect, explaining only 0.3% of the phenotypic variation. Also, no apparent interaction or correlation between the transcriptome and metabolome effects were observed, indicating that only the main additive effects of these omics contribute to COVID-19.

## Discussion

We applied statistical genetics models to study the effect of multiple omics on COVID-19. A single layer of omics can only provide limited insights into the biological mechanisms of a disease, implying consideration of several biological data is substantially helpful to fully dissect the complex molecular processes involved in disease development^17,18^. The complementary or antagonistic effects of biological signatures such as DNA, RNA, proteins, and metabolites on the development of complex traits or diseases have been studied^12^. However, previous COVID-19 studies have mainly investigated the contribution of host genetic risk factors^7,24^ and ignoring the potential role of other biological aspects, namely the transcriptome, metabolome, and exposome. Therefore, we employed a comprehensive integrative multi-omics analysis of COVID-19, incorporating four distinct omics layers involving the composite structure of SNP effects (genome), imputed gene expression level (transcriptome), metabolites (metabolome) and environmental features (exposome). Notably, we explored the variance components of COVID-19, specifically partitioning them into additive and non-additive omics effects, including interactions and correlations between omics layers.

We first systematically analysed the contribution of individual omics to COVID-19 by applying a single-omics model. Thus, the exposome and transcriptome (estimated using the coronary artery model) explained 3–4% of the variance in COVID-19, which is almost double the variance captured by the genomic (2.5%) and metabolomic (2%) effects. This has been demonstrated in omics-exposome models (i.e. simultaneous fitting of individual omics to the exposome), where the genome and metabolome shrink significantly when fitting with the exposome. This may highlight the role of the environment in mediating genomic and metabolomic impacts on COVID-19. In contrast, the transcriptome-induced phenotype variation remains stable. In particular, coronary arteries showed a strong COVID-19 expression signal in the tested tissue models used for transcriptomic analysis. Supporting evidence suggests that the relative expression of SARS-CoV-2 entry genes, including angiotensin-converting enzyme 2 (*ACE2*) and Basigin (*BSG*), is prominent in the endothelial layer of vascular tissues, including coronary arteries^62,63^. *ACE2* and *BSG* are surface receptors that facilitate viral uptake by host cells, and their expression increases with age^62,64,65^. As the current study involved UKB cohort participants aged 40–69, this may be one of the factors contribute to the significant transcriptome effect on COVID-19 in this age group. Single-omics analysis highlights the individual contribution of omics components in COVID-19 susceptibility, with certain tissues and omics data having a greater impact on phenotypic variation than others.

The analysis also revealed that the exposome has a strong signal for COVID-19, not only by itself, but also by modulating other omics layers. The study specifically examined the global influence of dietary intake, medical conditions (cancer, diabetes, asthma, etc.), and sociodemographic as exposome characteristics. Thus, combined effects account for a substantial proportion of phenotypic variation, highlighting the contribution of exogenous actors to the pathophysiology of COVID-19. Observational studies also support this, and the analysis of individual exposures (regardless of overall environmental determinants) demonstrate a potential role for the progression of COVID-19 ^66–68^. Collectively, the findings suggest that the exposome plays a crucial role in shaping disease phenotypes, and considering multiple molecular layers simultaneously can enhance our understanding of the mechanisms underlying COVID-19.

Through mediation analysis, study examined the significant role of exposomic factors in mediating the relationship between genomic, transcriptomic, and metabolomic influences on COVID-19 susceptibility^69^. The substantial mediation of genomic effects by exposomic factors underscores the importance of environmental and lifestyle factors in shaping genetic predispositions to COVID-19^70^. This finding emphasizes the complex interplay between genetic susceptibility and external influences, suggesting that interventions targeting environmental factors could potentially mitigate genetic risks associated with COVID-19^71^. Similarly, the significant mediation of metabolomic effects by exposomic factors further illustrates how environmental exposures can modify metabolic responses, reinforcing the crucial role of external factors in understanding COVID-19 susceptibility^72^. This highlights the need for a further analysis to explore how specific genetic and metabolic factors are mediated by environmental factors, aiding in the development of targeted strategies to avoid or modify external risk factors. Conversely, the relatively minor mediation of transcriptomic effects by exposomic factors suggests a more limited role of environmental factors in modulating transcriptomic pathways, particularly in coronary tissues. This finding indicates that while exposomic factors substantially influence genomic and metabolomic pathways, their impact on transcriptomic pathways may be less pronounced. It is possible that the transcriptomic response in specific tissues, such as the coronary artery, may be more directly regulated by intrinsic genetic and cellular mechanisms rather than external environmental factors. These results underscore the need for further research to elucidate the specific mechanisms through which exposomic factors influence transcriptomic responses and to explore potential tissue-specific interactions that may contribute to COVID-19 susceptibility. Overall, the results from the omics-exposome model and mediation analysis highlighted the critical role of the exposome in COVID-19.

It’s worth mentioning that the results of the study might not apply broadly to other ethnic groups since the study specifically used data from White British population only. Evidence shows that genetic variations can differ significantly between populations due to a combination of biological and environmental elements^73,74^. Genetic variants associated with phenotypes could vary in allele frequencies and effects across ancestral backgrounds^75^. This variation can affect gene-environment interactions, implying that the way genes and the environment interact plays a substantial role in determining an individual’s susceptibility or resistance to diseases such as COVID-19. Focusing on a single ethnic group, we risk overlooking these crucial differences, potentially resulting in an incomplete understanding of the biological mechanisms underlying COVID-19. Therefore, it is advised to conduct studies involving diverse populations to better understand the etiology of COVID1-19.

In addition to these generalizability concerns, the study has specific limitations. First, not all COVID-19 determinants were examined in the exposome analysis; however, considering existing knowledge, potential predictors consisting of 43 exposome features (e.g., medical conditions) were comprehensively evaluated. Second, another limitation arises from the accuracy constraints of the elastic-net model used for transcriptome imputation, as evidenced by significant gene expression being imputed in only a few tissues. Third, while the study investigated how exposomic features mediate the effects of the genome, transcriptome, and metabolome on COVID-19, it did not account for potential interaction effects between these omics layers in the mediation analysis. This is primarily because imputation generates predictions rather than direct measurements of gene expression, which can introduce biases in identifying genuine interactions^76,77^. As result, we restricted the analysis considering main effects to minimize imputation errors that could be amplified during interaction analysis. Lastly, the current integrative multi-omics approach does not include proteomic information. Given that the proteome is a key component of the omics landscape, future research incorporating proteomics-based methods will be crucial for further illuminating the variations in the clinical presentation of COVID-19.

Despite these limitations, the study has several notable strengths. First, the study integrated multi-omics data, including genome, transcriptome, metabolome, and exposome, each provides information on unique aspects of COVID-19. Bringing them together further provides new insights that cannot be obtained when analysing each of them independently, thereby helping to elucidate the underlying mechanisms of the disease. In other words, the study was able to reveal the interplay between molecular features, thus providing a new dimension for further understanding of biological clues. Second, in addition to the main omics effects, the study was able to dissect non-additive effects arising from complex interactions between omics layers, such as interaction and correlation effects. Furthermore, methodologically, this study applied novel linear mixed models (GREML and COR-GREML), which can accurately and efficiently integrate and interpret multidimensional omics data.

In conclusion, our study showed that an integrated analysis of COVID-19 using multi-omics data revealed the potential contribution of each layer of omics data to the disease. In particular, exposomes and transcriptomics appear to have independent contributions and explained considerable amount of variation in the clinical presentation of COVID-19. In contrast, the effects of genomics and metabolomics were small, and even adding the exposome into the model significantly attenuated the phenotypic variance explained by these two layers. We also observed a strong mediation effect of exposome on genome and metabolome effects on COVID-19, but a relatively weak mediating effect on transcriptomic influences. The results of study suggest that efficiently analysing and considering additional omics data (e.g., epigenome and proteome) may provide a more comprehensive biological insights into the variation of COVID-19 clinical presentation.

## Electronic supplementary material

Below is the link to the electronic supplementary material.

Supplementary Material 1

## Data Availability

The data used for this study are available from the UKB under an approved data request (https://www.ukbiobank.ac.uk/). Used tools along with code with related files can be accessed from the following web-resources.- MTG2 https://sites.google.com/site/honglee0707/mtg2 or from https://github.com/honglee0707/IGE.- PLINK https://www.cog-genomics.org/plink/. - MetaXcan https://github.com/hakyimlab/MetaXcan. - GCTA https://yanglab.westlake.edu.cn/software/gcta/. - LDSC https://github.com/bulik/ldsc.

## Abbreviations

ACME: Average causal mediation effect (ACME)
A_T_: Imputed transcriptome from adipose-visceral
B_T_: Imputed transcriptome from whole blood
CORE-GREML: Covariance between random effects-genome-based restricted maximum likelihood
COVID-19: Coronavirus disease 2019
C_T_: Imputed transcriptome from coronary arteries
DE: Direct effect
E: Exposome
ERM: Exposomic relationship matrix
G: Genome
GREML: Genome-based restricted maximum likelihood
GRM: Genetic relationship matrix
M: Metabolome
MRM: Metabolomic relationship matrix
M_T_: Imputed transcriptome from muscle skeletal
MV: Mediator variable
QC: Quality control
SNPs: Single nucleotide polymorphisms
SNP-h^2^: SNP-based heritability
S_T_: Imputed transcriptome from spleen
T: Transcriptome
TRM: Transcriptomic relationship matrix
UKB: UK Biobank
