# Supplementary material for: Integrative multi-omics analysis to gain new insights into COVID-19
Source: Sci Rep. 2024 Nov 30;14:29803. doi: 10.1038/s41598-024-79904-z (PMC11608341; doi:10.1038/s41598-024-79904-z)
Supplement: Supplementary file 1 — Supplementary Material 1 [file 41598_2024_79904_MOESM1_ESM.docx]

**Integrative multi-omics analysis to gain new insights into COVID-19**

Setegn Eshetie^1,2,3,4*^, Karmel W. Choi^5, 6^, Elina Hyppönen^1,4,7^, Beben Benyamin^1,2,4^, S. Hong Lee^1,2,4^

^1^Australian Centre for Precision Health, University of South Australia, SA, 5000, Australia.

^2^UniSA Allied Health and Human Performance, University of South Australia, SA, 5000, Australia.

^3^Department of Medical Microbiology, College of Medicine and Health Sciences, University of Gondar, Gondar, 196, Ethiopia.

^4^South Australian Health and Medical Research Institute (SAHMRI), University of South Australia, Adelaide, SA, 5000, Australia
^5^Center for Precision Psychiatry, Department of Psychiatry, Massachusetts General Hospital, Boston MA, USA

^6^Psychiatric & Neurodevelopmental Genetics Unit, Center for Genomic Medicine, Massachusetts General Hospital, Boston MA, USA

^7^UniSA Clinical and Health Sciences, University of South Australia, SA, 5000, Australia.

*Correspondence:

[kebsy003@mymail.unisa.edu.au](mailto:kebsy003@mymail.unisa.edu.au)

# **Supplementary file**

## Supplementary note 1

### Understanding of Multi-Omics Data used in the study

High-throughput technologies developed in recent years have made it possible to detect molecular features, namely gene transcripts, proteins, and metabolites, thereby helping to reveal molecular mechanisms under specific biological conditions. According to different levels of molecular characteristics, omics data can be divided into genomics, epigenomics, transcriptomics, proteomics, and metabolomics. In addition to primary molecular signatures, exposome signatures are also omics-scale characterization of nongenetic drivers of complex traits. Together with other omics, it defines the phenome of the individual. This study specifically utilized four omics layers, namely genome, transcriptome, metabolome, and exposome.

#### Genomics

Genomic approaches focus on the analysis of the constellation of DNA sequence (genome). Differences in DNA sequence between individuals, known as genetic variation, contribute to genetic and phenotypic diversity in populations by altering chromatin structure and modification, gene expression profiles, protein product structure, and/or metabolite abundance. Common techniques for characterizing genomic information include whole genome sequencing (WGS), whole exome sequencing (WES), and targeted sequencing and genotyping using whole genome oligonucleotide arrays. These techniques provide genotypes enriched for genetic variation (SNPS). While the most common type of genetic variations are SNPs, the genotype platforms also allow the characterization of other genetic variations such as insertions, deletions, and copy number variations, and etc. By quantifying the genotypes of genetic variants across the genome, researchers are able to perform a variety of analyzes including single variant assessment, multivariate aggregate analysis, epistasis studies of genetic variants involving multiple genes or regions, and interaction analysis with environmental factors.

#### Transcriptomics

RNA expression levels contain the genetic information transcribed from the genome; the collection of all RNA transcripts constitutes the transcriptome. Among RNA molecules, messenger RNAs (mRNAs) have attracted intense interest because they are intermediate components bridging DNA and proteins. Quantification of mRNA molecules provides a measure of gene expression, which can be achieved through the ensemble of all RNA sets by high-throughput technologies such as high-throughput gene expression microarrays or RNA sequencing (RNA-seq). Abnormal gene expression levels have been found to be the root of many diseases, helping to elucidate the etiology and molecular mechanisms of diseases. In this study, we imputed tissue-specific gene expression levels from genomic data, as transcriptome data in the UKB were not yet fully available.

#### Metabolomics

The metabolome refers to the complement of endogenous and exogenous low-molecular-weight molecules present within a biological system (such as a cell, tissue, organ, or organism) under a specific developmental or pathological state. The metabolome can reflect dynamic phenotypes, which are associated with psychological or pathological changes in the human body. Metabolomic profiles can be characterized by gas chromatography-mass spectrometry (GC-MS), liquid chromatography-mass spectrometry, and proton nuclear magnetic resonance (proton NMR) spectroscopy. Possible samples for the measurement of metabolites are in vitro cultures , supernatants, tissue extracts and biological fluids, especially urine and plasma.

#### Exposomics

External factors/environmental exposures or biological responses can be considered to define the scope of the exposome. Environmental factors are the entire set of all external exposures, ranging from individual level factors (e.g. smoking, radiation, physical activity, infectious agents, stress and etc) to population-based exposures (climate, air quality, social capital, urban/rural environment and etc). The concept of biological responses include metabolic changes, protein modifications, DNA mutations and adducts, epigenetic alterations, perturbations of the microbiome, disease and etc.

## **Supplementary note 2**

### **How to access COVID-19 phenotypic data from UK Biobank data?**

UK Biobank (UKBB) is an extensive biomedical database providing resources including genetic, phenotypic, and health-related data. The data comes from nearly 500,000 participants in England, Scotland and Wales aged 40-69. The database provides updated and supplementary data for researchers who are registered and approved to conduct research to address life-threatening health problems. Today, COVID-19 is one of the leading causes of morbidity and mortality and data on COVID-19 is now available at UKB and regularly augmented.

### **COVID-19 data types**

UKBB provides five COVID data types and are available on the data portal, such as 1) diagnostic COVID-19 test data, 2) COVID-19 miscellaneous data, 3) Death Register, 4) Hospital Impatient data, and 5) Primary Care (GP) data.

#### **Diagnostic COVID-19 test data**

Table S1 summarizes the number of COVID-19 positive and negative tests from three countries (UK, Scotland, and Wales) as March 2022 since the outbreak in December 2019. Current data shows that a total of 434, 119 people have been tested for COVID-19, of which nearly 9.5% (40,949 people) have tested positive.

#####

#### **COVID-19 miscellaneous**

Previous GWAS suggest that the ABO blood group system is significantly associated with COVID-19 susceptibility or severity. According to current UKBB statistics, nearly 43% (211,381) of UK participants have the OO blood group genotype, followed by AO, 36% (175,147) and BO, 9% (44,039) (Figure S1)

#### **Death register**

The UKBB death registry shows the date of death (3797 rows) and the primary and contributory causes of death, coded using the ICD-10 system (94440 rows). The format and structure of the death registration data is also detailed in the following pdf file (<https://biobank.ndph.ox.ac.uk/showcase/ukb/docs/DeathLinkage.pdf>)

#### **Hospital inpatient data**

These data provide information on hospital inpatient data available in the UKBB.

The hospital inpatient data includes:

- **hesin.text**: This is the overall master table, providing information on inpatient care events in England, Wales and Scotland (but currently excluding psychiatry and maternity inpatient events in Scotland), including details on admissions and discharge, the type of episode. This also includes, where applicable, how an episode fits into a hospital spell.
- **hesin_diag.txt**: Diagnostic codes (ICD-9 or ICD-10) associated with hospitalisation in England, Wales and Scotland (but currently excluding psychiatry and obstetric disorders in Scotland).
- **hesin_oper.txt**: Operational and procedural codes related to inpatient care (OPCS-3 or OPCS-4) (but currently excluding psychiatric and obstetric events in Scotland).
- **hesin_critical.txt:** Contains more information about hospital events requiring treatment in the intensive care unit. For instance, it gives the number of days of (basic & advanced) cardiac and respiratory support received by a patient.
- **hesin_psych.txt**: A sibling table to ‘hesin.txt’ containing data-fields (currently for England and Wales only) relating to administrative aspects of inpatient episodes of care for those Records relating to psychiatry, such as the history of psychiatric care and the legal status of the admission. Psychiatric diagnoses and procedures are contained in the ‘hesin_diag.txt’ and ‘hesin_oper.txt’.
- **hesin_maternity.txt**: A sibling table to HESIN containing data-fields (currently for England and Wales only) specifically relating to maternity inpatient episodes of care, such as antenatal information and whether anaesthetic was administered, etc. Diagnoses and procedures relating to maternity episodes are contained in the ‘hesin_diag.txt’ and ‘hesin_oper.txt’.
- **hesin_delivery.txt**: Information regarding a child born as a result of a ‘hesin_maternity.text’ record where a birth resulted, such birth weight, delivery method and place, and the status of the baby.

**NB**: For detail understanding follow the following links (<https://biobank.ndph.ox.ac.uk/showcase/ukb/docs/HospitalEpisodeStatistics.pdf>) and (<https://biobank.ndph.ox.ac.uk/ukb/ukb/docs/HESDataDic.xlsx>)

#### **Primary Care (GP) data**

GP data provides information on clinical events and prescription records for approximately 409,000 participants in England, with data from Scotland and Wales to be made available in due course.

There are two system suppliers for GP data;

1. TTP data system supplier (<https://www.tpp-uk.com/>)

- covid19_tpp_gp_clinical (clinical data from GP practices)
- covid19_tpp_gp_scripts (drug data from GP practices )

1. EMIS data system supplier (<https://www.emishealth.com/>)

- covid19_emis_gp_clinical (clinical data from GP practices)
- covid19_emis_gp_scripts (drug data from GP practices)

COVID-19 GP data contains coded clinical events (such as diagnosis, medical history, symptoms, laboratory results and procedures), prescriptions issued by GPs and a range of administrative codes (like referrals to specialist hospital clinics).

The details are provided in [resource 3151](https://biobank.ndph.ox.ac.uk/showcase/refer.cgi?id=3151), and [resource 951](https://biobank.ndph.ox.ac.uk/showcase/refer.cgi?id=951) (numeric values in TPP's clinical data)

**Table S1: The number COVID-19 positive and negative test results from UK**

| Source | Positive | Negative | Total |
| --- | --- | --- | --- |
| England | 37612 | 329719 | 367331 |
| Scotland | 2025 | 42685 | 44710 |
| Wales | 1312 | 20766 | 22078 |
| Total | 40949 | 393170 | 434119 |


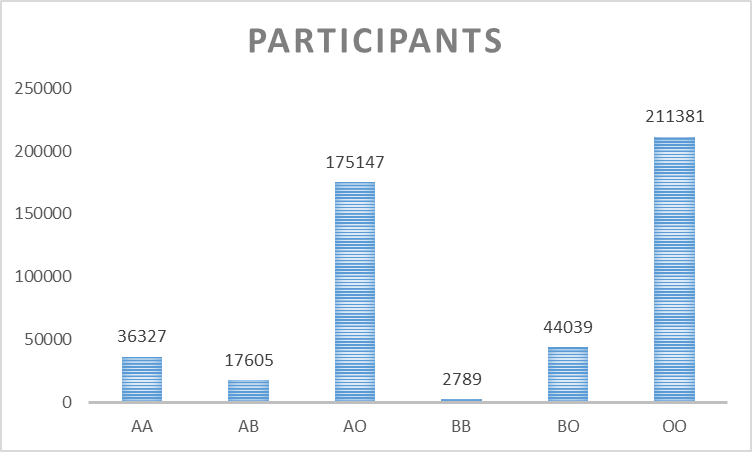


Figure S1: Distribution of ABO blood group in UK Biobank cohort.

## **Supplementary note 3**

### **Transcriptomic imputation using elastin net model**

Elastic net models consist of approximately 200000 HapMap2 SNPs from European tracks. Therefore, the current gene expression imputation analysis was based on Elastic net models, particularly using 18 tissue models (Table S1).

#### Table S2: Imputed tissue-specific gene expression levels based on COVID-19 genotypic data (N = 107857)

| Tissues | Number of genes with imputed gene expression | Percentage of genes with expression levels | Percentage of SNPs used in the model |
| --- | --- | --- | --- |
| Whole blood | 7232 | 99.8% (7232/7252) | 90% |
| Lung | 7935 | 99.6% (7935/7969) | 90% |
| Esophagus Mucosa | 8490 | 99.7% (8490/8521) | 90% |
| Coronary artery | 4026 | 99.6% (4026/4046) | 90% |
| Kidney Cortex | 1634 | 99.7% (1634/1642) | 90% |
| Liver | 3751 | 99.4% (3751/3773) | 90% |
| Transformed B-lymphocyte | 2893 | 99.7% (2893/2904) | 90% |
| Pancreas | 5875 | 99.7% (5875/5896) | 90% |
| Spleen | 5741 | 99.5% (5741/5774) | 90% |
| Adipose Subcutaneous | 8613 | 99.6% (8613/8650) | 90% |
| Artery Aorta | 7571 | 99.6% (7571/7599) | 90% |
| Heart Atrial Appendage | 6619 | 99.7% (6619/6641) | 90% |
| Adipose Visceral Omentum | 7312 | 99.6% (7312/7340) | 90% |
| Brain Cortex | 5470 | 99.5% (5470/5500) | 90% |
| Brain Hippocampus | 3664 | 99.4% (3664/3688) | 90% |
| Brain Hypothalamus | 3631 | 99.4% (3631/3652) | 90% |
| Colon Sigmoid | 6144 | 99.5% (6144/6173) | 90% |
| Muscle skeletal | 7566 | 99.8 % (7566/7583) | 90% |

#### Table S3: Evaluation of the effect of tissue-specific transcriptomic profile for COVID-19.

| Tissues | Adjusted r-squared | P-value |
| --- | --- | --- |
| *Whole blood* | 0.0033 | 2.4e-03* |
| *Lung* | 0.0027 | 4.6e-02 |
| *Esophagus Mucosa* | 0.0025 | 4.9e-02 |
| *Coronary artery* | 0.0347 | 1.2e-239* |
| *Kidney Cortex* | 0.001 | 5.9e-02 |
| *Muscle skeletal* | 0.005271 | 6.24e-06* |
| *Liver* | 0.0018 | 5.2e-02 |
| *Transformed B-lymphocyte* | 0.0015 | 5.7e-02 |
| *Pancreas* | 0.002 | 5.1e-02 |
| *Spleen* | 0.01162 | 3.9e-27* |
| *Adipose Subcutaneous* | 0.0025 | 4.8e-02 |
| *Artery Aorta* | 0.0028 | 4.7e-02 |
| *Heart Atrial Appendage* | 0.0027 | 4.8e-02 |
| *Adipose Visceral Omentum* | 0.003567 | 1.2e-03* |
| *Brain Cortex* | 0.0031 | 4.5e-02 |
| *Brain Hippocampus* | 0.0021 | 4.8e-02 |
| *Brain Hypothalamus* | 0.0033 | 4.4e-02 |
| *Colon Sigmoid* | 0.002 | 5.3e-02 |

### Table S4: Regression analysis on the effect of exposomic features on COVID-19 Susceptibility.

### *The table presents exposomic features alongside their corresponding beta coefficients, standard errors, and P-values.*

| Exposomic features | Beta | Std_Error | P_value |
| --- | --- | --- | --- |
| sex | 0.021772 | 0.011636 | 6.13E-02 |
| year_of_birth* | 0.008435 | 0.003536 | 1.71E-02 |
| age** | -0.01375 | 0.003541 | 1.03E-04 |
| tdi*** | 0.010342 | 0.001117 | 2.05E-20 |
| smoking_status | -0.01045 | 0.0046 | 2.31E-02 |
| alcohol_status | 0.016928 | 0.008182 | 3.86E-02 |
| bmi | -0.0113 | 0.006387 | 7.68E-02 |
| edu_level*** | -0.0108 | 0.000641 | 1.54E-63 |
| pc1 | -0.00429 | 0.002023 | 3.37E-02 |
| pc2 | 0.000173 | 0.002098 | 9.34E-01 |
| pc3 | 0.001418 | 0.002029 | 4.84E-01 |
| pc4 | 0.004353 | 0.00151 | 3.95E-03 |
| pc5* | 0.001948 | 0.000686 | 4.52E-03 |
| pc6 | 0.000905 | 0.001929 | 6.39E-01 |
| pc7 | 0.001145 | 0.001737 | 5.10E-01 |
| pc8 | -0.00529 | 0.001723 | 2.13E-03 |
| pc9 | -0.00065 | 0.000757 | 3.91E-01 |
| pc10*** | 0.007183 | 0.001513 | 2.05E-06 |
| Waist circumference | 0.00133 | 0.000613 | 3.00E-02 |
| Hip circumference | -0.00147 | 0.000809 | 6.92E-02 |
| standing height | -0.00904 | 0.002181 | 3.41E-05 |
| sitting height | 0.001852 | 0.001283 | 1.49E-01 |
| weight | 0.006404 | 0.002148 | 2.87E-03 |
| Bread intake** | 0.001788 | 0.000379 | 2.34E-06 |
| cereal intake | 0.001035 | 0.000811 | 2.02E-01 |
| cooked vegetable intake | -0.00028 | 0.001342 | 8.33E-01 |
| dried fruit intake | -0.00055 | 0.000959 | 5.65E-01 |
| fresh fruit intake | 0.001469 | 0.00126 | 2.44E-01 |
| salad/ raw vegetable intake | 0.001029 | 0.001053 | 3.29E-01 |
| tea intake | 0.001254 | 0.000874 | 1.51E-01 |
| water intake** | -0.00299 | 0.00084 | 3.66E-04 |
| beef intake | 0.006254 | 0.004431 | 1.58E-01 |
| cheese intake | -0.00637 | 0.003 | 3.39E-02 |
| lamb/mutton intake*** | -0.02395 | 0.005243 | 4.92E-06 |
| oily fish intake*** | -0.01854 | 0.003516 | 1.35E-07 |
| pork intake | 0.003284 | 0.005034 | 5.14E-01 |
| poultry intake*** | 0.018517 | 0.003854 | 1.56E-06 |
| cancer diagnosed by doctor | 0.02662 | 0.011188 | 1.74E-02 |
| diabetes diagnosed by doctor | -0.01601 | 0.014174 | 2.59E-01 |
| blood clot, deep vein thrombosis (DVT), bronchitis, emphysema, asthma, rhinitis, eczema, allergy diagnosed by doctor, | 0.000906 | 0.000433 | 3.64E-02 |
| diastolic blood pressure automated reading | 0.000118 | 0.000432 | 7.84E-01 |
| pulse rate automated reading*** | 0.001366 | 0.00029 | 2.39E-06 |
| systolic blood pressure automated reading | -0.00013 | 0.000235 | 5.91E-01 |


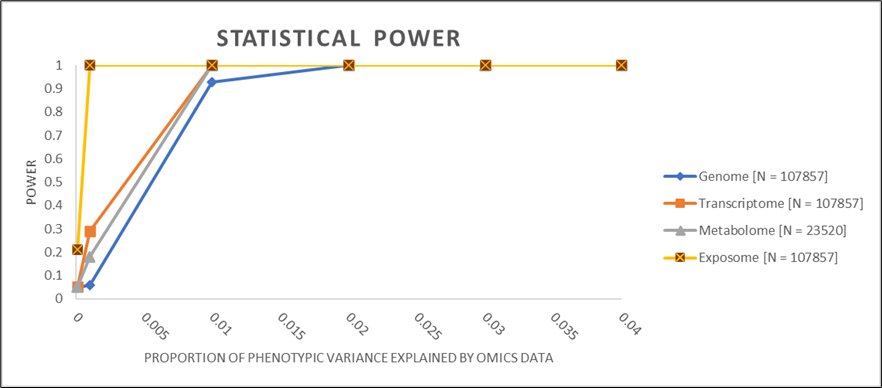


Figure S2: The statistical power estimation for the proportion of phenotypic variance in COVID-19 explained by genomic, transcriptomic, metabolomic, and exposomic data, given the sample size of this study.

*Power estimation was conducted using five key parameters as required by the GCTA power calculator (https://shiny.cnsgenomics.com/gctaPower/): 1) sample sizes for each omics dataset, metabolome data with N = 23,520 and the other omics datasets with N = 107,857; 2) a significance threshold (α) set at 0.05; 3) the proportion of phenotypic variance explained by each dataset, ranging from 0 to 0.04; 4) the proportion of cases in the sample (k = 0.19); and 5) the variance of off-diagonal elements of the relationship matrices. The variance of the off-diagonal elements for the genetic relationship matrix (GRM) was estimated to be 2.0e-05. In addition, the variances of the off-diagonal elements for other omics matrices were calculated as follows: 3.4e-04 for the transcriptomic relationship matrix (TRM), 4.0e-03 for the metabolomic relationship matrix (MRM), and 2.3e-02 for the exposomic relationship matrix (ERM).*

*The x-axis represents the proportion of phenotypic variance explained, while the y-axis indicates statistical power (ranging from 0 to 1). Each omics component is depicted by a distinct color-coded line.*

## **Supplementary note 4:** Variance component analysis

### Table S5: The proportion of COVID-19 phenotypic variance considering omics-exposome interactions.

| Model | Layers | Proportion of phenotypic variance | Standard error | P-value |
| --- | --- | --- | --- | --- |
| *Y=G+E+GxE+ꜫ* | *Genome* | 0.0104 | 0.003 | 5.6e-04 |
|  | *Exposome* | 0.0384 | 0.008 | 2.2e-06 |
|  | *Interaction* | 0.0014 | 0.007 | 8.50e-01 |
| *Y=T+E+TxE+ꜫ* | *Adipose viscera* | 0.0012 | 0.0009 | 1.80e-01 |
|  | *Exposome* | 0.0385 | 0.0081 | 2.74e-06 |
|  | *Interaction* | -0.0003 | 0.0046 | 1.00e+00 |
|  | *Coronay artery* | 0.0307 | 0.0015 | 8.88e-83 |
|  | *Exposome* | 0.0372 | 0.0078 | 2.54e-06 |
|  | *Interaction* | 0.0004 | 0.0038 | 9.20e-01 |
|  | *Muscle skeletal* | 0.0031 | 0.001 | 1.90e-03 |
|  | *Exposome* | 0.0386 | 0.0081 | 2.59e-06 |
|  | *Interaction* | -0.0136 | 0.0044 | 1.00e+00 |
|  | *Spleen* | 0.0048 | 0.001 | 2.2e-06 |
|  | *Exposome* | 0.0384 | 0.008 | 2.2e-06 |
|  | *Interaction* | 0.0011 | 0.0043 | 8.10e-01 |
|  | *Whole blood* | 0.0015 | 0.0009 | 9.50e-02 |
|  | *Exposome* | 0.0385 | 0.0081 | 2.74e-06 |
|  | *Interaction* | -0.0016 | 0.0045 | 1.00e+00 |
| *Y=M+E+MXE+ꜫ* | *Metabolome* | 0.0036 | 0.0013 | 5.60e-03 |
|  | *Exposome* | 0.0394 | 0.0086 | 6.04e-06 |
|  | *Interaction* | -0.0034 | 0.0041 | 1.00e+00 |

*G: genome, T: transcriptome profile of five tissues (coronary artery, spleen, adipose visceral, muscle skeletal and whole blood), M: metabolome and E: exposome. GxE: genome-exposome interaction, TxE: transcriptome-exposome interaction, and MxE: metabolome-exposome interaction.*

### Table S6: The proportion of COVID-19 phenotypic variance considering omics-exposome correlations.

| Model | Layers | Proportion of phenotypic variance | Standard error | P-value |
| --- | --- | --- | --- | --- |
| *Y=G+E+r_G,E_+ Ε* | *Genome* | *0.011* | *0.003* | *8.4e-04* |
|  | *Exposome* | *0.039* | *0.008* | *2.8e-06* |
|  | *Correlation* | *-0.33* | *0.80* | *6.8e-01* |
| *Y=T+E+ r_T,E_ +ꜫ* | *Adipose viscera* | *0.0013* | *0.0009* | *1.63e-01* |
|  | *Exposome* | *0.039* | *0.0082* | *2.5e-06* |
|  | *correlation* | *-0.96* | *0.91* | *0.30* |
|  | *Coronary artery* | *0.031* | *0.0016* | *0.00e+00* |
|  | *Exposome* | *0.0374* | *0.0079* | *2.54e-06* |
|  | *Correlation* | *-0.15* | *0.21* | *4.9e-01* |
|  | *Muscle skeletal* | *0.0031* | *0.0009* | *1.38e-03* |
|  | *Exposome* | *0.039* | *0.0082* | *2.2e-06* |
|  | *Correlation* | *-0.36* | *0.60* | *5.6e-01* |
|  | *Spleen* | *0.0047* | *0.001* | *7.7e-06* |
|  | *Exposome* | *0.0383* | *0.008* | *1.7e-06* |
|  | *Correlation* | *0.17* | *0.47* | *7.3e-01* |
|  | *Whole blood* | *0.0014* | *0.00089* | *1.0e-01* |
|  | *Exposome* | *0.039* | *0.0081* | *2.0e-06* |
|  | *Correlation* | *-0.12* | *0.78* | *8.8e-01* |
| *Y=M+E+ r_M,E_ +ꜫ* | *Metabolome* | *0.0035* | *0.0013* | *5.60e-03* |
|  | *Exposome* | *0.0391* | *0.0085* | *4.0e-06* |
|  | *Correlation* | *0.18* | *0.30* | *5.5e-01* |

*G: genome, T: transcriptome profile of five tissues (coronary artery, spleen, adipose visceral, muscle skeletal and whole blood), M: metabolome and E: exposome. r_G,E_ genome-exposome correlation, r_T,E_: transcriptome-exposome correlation, and r_M,E_: metabolome-exposome correlation.*

### Table S7: Comparison of individual omics models with models including exposomic effects.

| Variance components | G | G and E |
| --- | --- | --- |
| σ^2^_g_/ σ^2^_y_ | 0.0251[0.02-0.031] | 0.0104[0.005-0.02] |
| σ^2^_e_/ σ^2^_y_ |  | 0.0384[0.03-0.06] |
| LKH | -55476.2273 | -53490.7848, p=0* |
|  |  |  |
| Variance components | ***T*** | ***T and E*** |
| σ^2^_t_/ σ^2^_y_ | 0.034[0.03-0.04] | 0.031[0.03-0.034] |
| σ^2^_e_/ σ^2^_y_ |  | 0.0372[0.022-0.053] |
| LKH | -55238.8707 | -53059.4217, p=0* |
|  |  |  |
| Variance components | ***M*** | ***M and E*** |
| σ^2^_m_/ σ^2^_y_ | 0.0194[0.015-0.025] | 0.0035[0.001-0.0061] |
| σ^2^_e_/ σ^2^_y_ |  | 0.0395[0.023-0.06] |
| LKH | -12002.6237 | -11666.9694, p=5.2e-148* |

*σ^2^_g_/ σ^2^_y:_ proportion of phenotypic variance explained by genome (G), σ^2^_e_/ σ^2^_y:_ proportion of phenotypic variance explained by exposome (E), σ^2^_t_/ σ^2^_y:_ proportion of phenotypic variance explained by transcriptome (T), σ^2^_m_/ σ^2^_y:_ Proportion of phenotypic variance explained by metabolome. The goodness-of-fit of omics models was assessed based on loglikelihood ratios (LKH).*

### Table S8: Comparison of individual omics models with pairwise-omics models in capturing phenotypic variance.

| Variance components | G | G and T |
| --- | --- | --- |
| σ^2^_g_/ σ^2^_y_ | 0.0251[0.02-0.031] | 0.0156 [0.008-0.02] |
| σ^2^_t_/ σ^2^_y_ |  | 0.0343 [0.03-0.038] |
| LKH | -55476.2273 | -54999.6138, p=0* |
|  |  |  |
| Variance components | ***T*** | ***G and T*** |
| σ^2^_t_/ σ^2^_y_ | 0.034[0.03-0.04] | 0.0156 [0.008-0.02] |
| σ^2^_g_/ σ^2^_y_ |  | 0.0343 [0.03-0.038] |
| LKH | -55238.8707 | -54999.6138, p=0* |
|  |  |  |
| Variance components | T | T and M |
| σ^2^_t_/ σ^2^_y_ | 0.034[0.03-0.04] | 0.0262[0.02-0.034] |
| σ^2^_m_/ σ^2^_y_ |  | 0.0190[0.01-0.02] |
| LKH | -55238.8707 | -11977.3456, p=0* |
|  |  |  |
| Variance components | M | T and M |
| σ^2^_m_/ σ^2^_y_ | 0.0194[0.015-0.025] | 0.0262[0.02-0.034] |
| σ^2^_t_/ σ^2^_y_ |  | 0.0190[0.01-0.02] |
| LKH | -12002.6237 | -11977.3456, p=0* |
|  |  |  |

*σ^2^_g_/ σ^2^_y:_ proportion of phenotypic variance explained by genome (G), σ^2^_t_/ σ^2^_y:_ proportion of phenotypic variance explained by transcriptome (T), σ^2^_m_/ σ^2^_y:_ Proportion of phenotypic variance explained by metabolome. The goodness-of-fit of omics models was assessed based on loglikelihood ratios (LKH).*

## Supplementary note 5

### The antagonistic interplay between genome and transcriptome in COVID-19

To better understand the complex interplay between different omics layers, we investigated the genome-transcriptome interaction in our multi-omics panel , using imputed transcriptome in coronary artery tissue. Our results revealed that the genome-transcriptome interaction (GxT) explained approximately 31% (SE=9%, P-value=6.1e-04) of the phenotypic variance in COVID-19, making it a critical factor in explaining most of the variation (Figure S2). Additionally, we observed a strong negative correlation between the genome and transcriptome effects of COVID-19 (r = -0.54 + 0.11, P-value = 3.1e-07), suggesting an antagonistic interplay between these two molecular layers that underlie the phenotypic variance in the disease. Importantly, we found that when jointly fitting with exposome, the estimates of genome-transcriptome correlation and interaction were not significantly altered, highlighting their robustness (Figure S3). These results underscore the importance of considering both genome-transcriptome correlation and interaction to fully comprehend the contributions of the different omics layers and their interplay in COVID-19.

Although coronary artery tissue was identified as the main contributing factor for COVID-19 (as shown in Figure 3), we also investigated the genome-transcriptome interplay using imputed transcriptome in other tissues such as spleen, musculoskeletal, adipose viscera, and whole blood. More specifically, using spleen tissue, the estimated GxT interaction was found to be significant after multiple testing, capturing 29% of the phenotypic variation (Table S8). We also observed a small GxT interaction signal (estimated at 2%) when using muscle tissue (Table S8), whereas no significant GxT interaction was detected when considering gene expression profiles of whole blood and adipose viscera. Furthermore, genome-transcriptome correlation for those tissues (spleen, musculoskeletal, adipose viscera, and whole blood) was not significant.


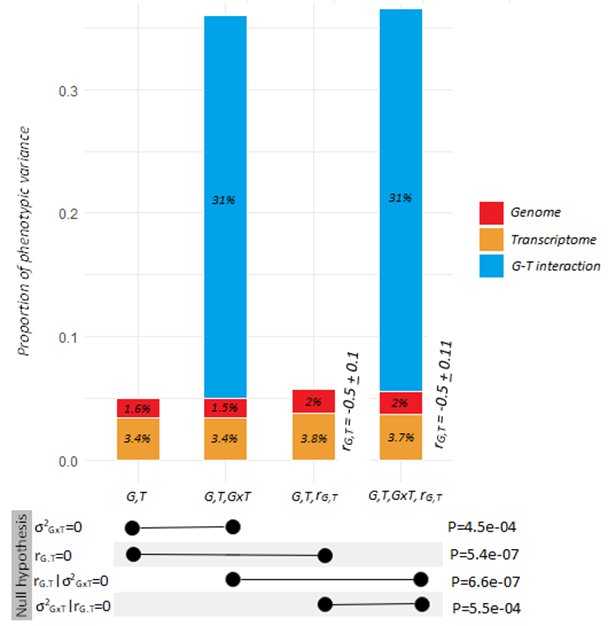


*Figure S3: Interplay between genome and coronary transcriptome in explaining COVID-19 phenotypic variance.*

*A multi-effects linear mixed model fitting genome and transcriptome (imputed in coronary artery tissue) was used to estimate the proportion of COVID-19 phenotypic variance explained by the main additive effects of genome (G), transcriptome (T), and interaction (GxT) and correlatin (r_G,T_) between genomic and transcriptomic effects on COVID-19. Four different models were used (plotted on x-axis) to estimate the proportion of COVID-19 phenotypic variance (plotted on y-axis): 1) G,T model includes the main additive effects of genome and transcriptome, 2) G,T,GxT model includes the interaction effects between genome and transcriptome in addition to the main additive effect, 3) G,T, r_G,T_ model includes the correlation between genome and transcriptome in addition to the main additive effect, and 4) G,T,GxT,r_G,T_ model includes both the interaction and correlation between between genome and transcriptome in addition to the main additive effects. To assess the model fit, likelihood ratio tests were performed, and p-values were plotted in the upset plot below the graph. The solid circles represent the comparison between the full model and the reduced model. A smaller p-value indicates a better fit of the full model.*

### Table S9: Proportion of COVID-19 phenotypic variance explained by multi-omics layers (genome, transcriptome and exposome).

| Model | Layers | Proportion of phenotypic variance | Standard error | P-value |
| --- | --- | --- | --- | --- |
| *Y=G+C_T_+ꜫ* | *Genome* | *0.0156* | *0.003* | *3.1e-07* |
|  | *Transcriptome* | *0.0343* | *0.002* | *1.98e-90* |
| *Y=G+C_T_+E+ ꜫ* | *Genome* | *0.003* | *0.003* | *3.2e-01* |
|  | *Transcriptome* | *0.031* | *0.002* | *5.9e-49* |
|  | *Exposome* | *0.0372* | *0.0078* | *2.54e-06* |
| *Y=G+C_T_+GXT+ꜫ* | *Genome* | *0.02* | *0.003* | *7.8e-11* |
|  | *Transcriptome* | *0.034* | *0.0016* | *1.98e-90* |
|  | *GxT* | *0.31* | *0.09* | *6.1e-04* |
| *Y=G+S_T_+GXT+ꜫ* | *Genome* | *0.02* | *0.003* | *7.8e-11* |
|  | *Transcriptome* | *0.005* | *0.001* | *8.4e-07* |
|  | *GxT* | *0.29* | *0.09* | *1.3e-03* |
| *Y=G+M_T_+GXT+ꜫ* | *Genome* | *0.022* | *0.003* | *1.0e-12* |
|  | *Transcriptome* | *0.003* | *0.001* | *2.8e-03* |
|  | *GxT* | *0.02* | *0.009* | *2.6e-02* |
| *Y=G+C_T_+GXT+E+ꜫ* | *Genome* | *0.003* | *0.003* | *3.2e-01* |
|  | *Transcriptome* | *0.031* | *0.002* | *5.9e-49* |
|  | *GxT* | *0.33* | *0.09* | *2.7e-04* |
|  | *Exposome* | *0.0372* | *0.0078* | *2.54e-06* |
| *Y=G+S_T_+GXT+E+ꜫ* | *Genome* | *0.006* | *0.003* | *4.5e-02* |
|  | *Transcriptome* | *0.004* | *0.001* | *2.1e-05* |
|  | *GxT* | *0.25* | *0.09* | *8.8e-03* |
|  | *Exposome* | *0.038* | *0.008* | *2.8e-06* |
| *Y=G+M_T_+GXT+E+ꜫ* | *Genome* | *0.008* | *0.003* | *7.7e-03* |
|  | *Transcriptome* | *0.002* | *0.001* | *2.8e-03* |
|  | *GxT* | *0.02* | *0.009* | *2.6e-02* |
|  | *Exposome* | *0.038* | *0.008* | *2.8e-06* |
| *Y=G+C_T_+r_G,T_+ Ε* | *Genome* | *0.02* | *0.003* | *1.8e-08* |
|  | *Transcriptome* | *0.037* | *0.002* | *2.55e-68* |
|  | *Correlation (G,T)* | *-0.54* | *0.11* | *2.6e-07* |

*Note: G: genome, C_T_: transcriptome [coronary artery], S_T_: transcriptome [Spleen], M_T_: transcriptome [Muscle skeletal], M: metabolome and E: exposome. GxT: genome-transcriptome interaction, r_G,T_: genome-transcriptome correlation.*


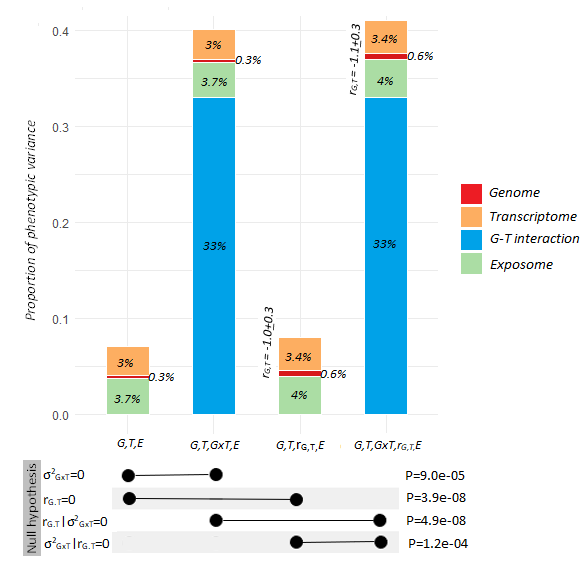


### Figure S4: Contribution of multi-omics (genome, transcriptome and exposome) in explaining COVID-19 phenotypic variance.

*A multi-effects linear mixed model fitting genome, transcriptome (imputed in coronary artery tissue) and exposome was used to estimate the proportion of COVID-19 phenotypic variance explained by the main additive effects of genome (G), transcriptome (T), exposome (E) and interaction (GxT) and correlatin (r_G,T_) between genomic and transcriptomic effects on COVID-19. Four different models were used (plotted on x-axis) to estimate the proportion of COVID-19 phenotypic variance (plotted on y-axis): 1) G,T, E model includes the main additive effects of genome and transcriptome, 2) G,T,GxT,E model includes the interaction effects between genome and transcriptome in addition to the main additive effect, 3) G,T, r_G,T_, E model includes the correlation between genome and transcriptome in addition to the main additive effect, and 4) G,T,GxT,r_G,T_,E model includes both the interaction and correlation between between genome and transcriptome in addition to the main additive effects. To assess the model fit, likelihood ratio tests were performed, and p-values were plotted in the upset plot below the graph. The solid circles represent the comparison between the full model and the reduced model. A smaller p-value indicates a better fit of the full model.*

### Table S10: Proportion of COVID-19 phenotypic variance explained by multi-omics layers (transcriptome, metabolome and exposome).

| Model | Layers | Proportion of phenotypic variance | Standard error | P-value |
| --- | --- | --- | --- | --- |
| *Y=T+M+ꜫ* | *Transcriptome* | *0.026* | *0.004* | *2.2E-10* |
|  | *Metabolome* | *0.019* | *0.0026* | *5.45E-20* |
| *Y=T+M+E+ꜫ* | *Transcriptome* | *0.024* | *0.004* | *4.24E-09* |
|  | *Metabolome* | *0.0034* | *0.001* | *8.9E-03* |
|  | *Exposome* | *0.039* | *0.009* | *5.6E-06* |
| *Y=T+M+TXM+ ꜫ* | *Transcriptome* | *0.032* | *0.004* | *8.8E-15* |
|  | *Metabolome* | *0.0094* | *0.0012* | *2.9E-14* |
|  | *TXM* | *-0.019* | *0.0019* | *1.00E+00* |
| *Y=T+M+TXM+ E+ ꜫ* | *Transcriptome* | *0.024* | *0.004* | *4.2E-09* |
|  | *Metabolome* | *0.0039* | *0.001* | *1.1E-04* |
|  | *TXM* | *-0.011* | *0.003* | *1.00E+00* |
|  | *Exposome* | *0.039* | *0.008* | *1.54E-06* |
| *Y=T+M+r_T,M_+ ꜫ* | *Transcriptome* | *0.026* | *0.004* | *2.2E-10* |
|  | *Metabolome* | *0.0189* | *0.0026* | *3.8E-13* |
|  | *Correlation* | *0.04* | *0.19* | *8.4E-01* |

*Note: T: transcriptome of coronary artery, M: metabolome and E: exposome. TxM transcriptome-metabolome interaction, r_T,M_: transcriptome-metabolome correlation.*
